# Molecular Indicators of Biomaterials Osteoinductivity - Cell Migration, BMP Production and Signalling Turns a Key

**DOI:** 10.1007/s12015-021-10300-0

**Published:** 2021-11-16

**Authors:** Krzysztof Łukowicz, Barbara Zagrajczuk, Jarosław Wieczorek, Katarzyna Millan-Ciesielska, Izabela Polkowska, Katarzyna Cholewa-Kowalska, Anna M. Osyczka

**Affiliations:** 1 Faculty of Biology, Institute of Zoology and Biomedical Research, Department of Biology and Cell Imaging, Jagiellonian University, 9 Gronostajowa St, 30-387 Krakow, Poland; 2 Faculty of Materials Science and Ceramics, Department of Glass Technology and Amorphous Coatings, AGH University of Science and Technology, 30 Mickiewicza Ave, 30-059 Krakow, Poland; 3 University Centre of Veterinary Medicine UJ-UR, University of Agriculture in Krakow, 24/28 Mickiewicza Ave., 30-059 Krakow, Poland; 4 Faculty of Medicine, Department of Pathomorphology, Jagiellonian University Medical College, 2 Jakubowskiego St., 30-688 Krakow, Poland; 5 Faculty of Veterinary Medicine, Department and Clinic of Animal Surgery, University of Life Sciences in Lublin, 13 Akademicka St., 20-950 Lublin, Poland

**Keywords:** Sol-gel bioactive glass/PLGA scaffolds, Human bone marrow stromal cells, Cell migration, BMP-related signalling, Osteogenesis, Ectopic bone formation

## Abstract

In this work we dissected the osteoinductive properties of selected, PLGA-based scaffolds enriched with gel-derived bioactive glasses (SBGs) of either binary SiO_2_-CaO or ternary SiO_2_-CaO-P_2_O_5_ system, differing in CaO/SiO_2_ ratio (i.e. high -or low-calcium SBGs). To assess the inherent ability of the scaffolds to induce osteogenesis of human bone marrow stromal cells (BMSC), the study was designed to avoid any osteogenic stimuli beyond the putative osteogenic SBG component of the studied scaffolds. The bioactivity and porosity of scaffolds were confirmed by SBF test and porosimetry. Condition media (CM) from BMSC-loaded scaffolds exhibited increased Ca and decreased P content corresponding to SBGs CaO/SiO_2_ ratio, whereas Si content was relatively stable and overall lower in CM from scaffolds containing binary SBGs. CM from cell-loaded scaffolds containing high-calcium, binary SBGs promoted migration of BMSC and BMP-response in reporter osteoblast cell line. BMSC culture on these scaffolds or the ones containing ternary, low-calcium SBGs resulted in the activation of BMP-related signaling and expression of several osteogenic markers. Ectopic bone formation was induced by scaffolds containing binary SBGs, but high-calcium ones produced significantly more osteoid. Scaffolds containing ternary SBGs negatively influenced the expression of osteogenic transcription factors and Cx43, involved in cell-cell interactions. High-calcium scaffolds stimulated overall higher Cx43 expression. We believe the initial cell-cell communication may be crucial to induce and maintain osteogenesis and high BMP signaling on the studied scaffolds. The presented scaffolds’ biological properties may also constitute new helpful markers to predict osteoinductive potential of other bioactive implant materials.

**Graphical Abstract:**

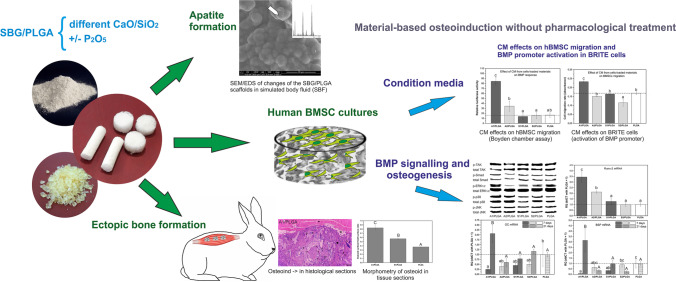

## Introduction

Nowadays, tissue engineering relies heavily on natural and artificial scaffolds that are expected to provide the environment suitable for stem cells differentiation into desired tissue phenotype. If cells populating the scaffold are required to differentiate into a given pathway it is of benefit to design biologically active scaffolds that, thanks to their inherent properties, are capable to induce and support cell differentiation. Such approach avoids complexities related to additional stem cell treatment and, considering clinical applications, it may markedly reduce the time required for biofabrication of appropriate cell-loaded material construct.

In bone tissue engineering bioactive glasses offer a promising and broadly used component of material coatings and scaffolds as they are known to form tight bonding with bone and they may induce and support osteogenesis of mesenchymal stem cells [1]. Incorporation of bioactive glasses into polymer matrix provides several advantages such as tailored bioactivity, biodegradability and mechanical properties [2–6]. Applying sol-gel technology to obtain bioactive glasses leads to yet another benefits such us mesoporous glass texture, highly-developed surface and exposition of siloxane groups on the material surface, suitable for hydroxyapatite nucleation [7–10]. Upon contact with physiological solutions bioactive glasses also release ions, such as calcium (Ca^2+^) and phosphate (PO_4_^3⁠−^) ions, which contribute to cell response [11–13].

It is now apparent that the chemical composition of the scaffold affects its physical properties and it may be especially important for 3D architecture of the scaffold that, in turn, affects cell attachment, proliferation and differentiation [2, 14–17]. Previously we showed that the composites obtained from PLGA and four chemically different gel-derived bioactive glasses from the SiO_2_–CaO/ ±P_2_O_5_ system that had been prepared in the form of thin films placed at the bottom of culture wells and used as BMSC growth surface, were capable to prompt osteogenesis of human bone marrow stromal cells (hBMSC) cultures without any additional osteogenic stimuli. That was partly dependent on the absence/presence of P_2_O_5_ in the composites chemical composition and direct cell-material contact, since treating cells with condition media from the composites was not as effective as the direct interaction of cells with the material surfaces. Aware that the osteoinductive material properties may be altered with the changes of material architecture [18, 19], we selected two composite materials of different CaO/SiO_2_ ratio, both with and without P_2_O_5_ to further explore their osteoinductive potential when prepared in the form of porous, 3D scaffolds. We sought to address the questions whether the endogenous expression of osteogenic bone morphogenetic proteins (BMP) and activation of BMP-dependent intracellular signalling is a general feature of osteoinductive materials and a key biological mechanism responsible for the osteoinduction in bone precursor cells regardless of the complexity of bioactive materials structure. We show that the 3D scaffolds made of PLGA enriched with high-calcium bioactive glass depleted of P_2_O_5_ are capable to induce human BMSC migration, BMP endogenous expression and intracellular signalling and maintain osteogenesis of long-term hBMSC cultures without any additional osteogenic stimuli, consistent with their ectopic bone formation abilities in a rabbit model.

## Materials and Methods

### Scaffolds Preparation and Characteristics

#### Bioactive Glass Preparation

Chemical compositions of the sol-gel derived glasses used in this work for the preparation of PLGA–based composite scaffolds are presented in Table 1. Gel-derived bioactive glasses (SBG) were ordered into two groups depending on their chemical compositions: the ones of the binary SiO_2_–CaO system were assigned to group I, and the others from the ternary SiO_2_–CaO–P_2_O_5_ system were assigned to group II. Tetraethyl orthosilicate (TEOS; Si(OC_2_H_5_)_4_), triethylphosphate (TEP; OP(OC_2_H_5_)_3_) (both Sigma-Aldrich, USA), and calcium nitrate tetra-hydrate (Ca(NO_3_)_2_·4H_2_O) (POCH, Poland) were used as main components in the sol-gel process. Solution of 1 M HCl (POCH, Poland) was used as a catalyst for the hydrolysis and condensation reactions. Formed gels were dried gradually in the temperature range 40–120 °C, and then subjected to heat treatment at 600 °C for 10 h (SiO_2_–CaO) or 700 °C for 20 h (SiO_2_–CaO–P_2_O_5_). Afterwards, the samples were milled and sieved to obtain bioactive glass powders with average particle diameter ca 45 μm.The textural characteristics of the obtained SBGs were reported previously [20].

**Table 1:** Chemical compositions of the SBGs and their CaO/SiO_2_ ratios

Material	Chemical composition (%mol)			CaO/SiO_2_ ratio
	SiO_2_	CaO	P_2_O_5_	
A1	40	60	–	1.50
S1	80	20	–	0.25
A2	40	54	6	1.35
S2	80	16	4	0.20

#### Composite Scaffolds Fabrication

PLGA (poly L-lactide-co-glycolide) was synthesized via a ring-opening process in the presence of low toxicity zirconium acetylacetonate as a copolymerization initiator [21]. The molar ratio of L-lactide to glycolide in the copolymer was 85:15; as determined by ^1^H nuclear magnetic resonance (NMR) and molecular masses of PLGA were: Mn = 80 kDa and Mw = 152 kDa. The composite scaffolds were fabricated by a solvent casting particulate leaching method (SCPL). The PLGA polymer was dissolved in dichloromethane (DCM, POCH, Poland) by continuous stirring for 24 h to obtain 10%w/v polymer solution. Then glass particles (21%vol.) were thoroughly dispersed in the polymer solution by stirring for the next 24 h. Afterwards sodium chloride particles (POCH, Poland, grain fraction 320–400 μm) were added into the SBG–PLGA suspension and poured onto Petri dishes. The amount of sodium chloride provided 85% porosity. NaCl particles were thoroughly mixed with SBG–PLGA suspension until most of the DCM had evaporated to obtain dense mixture, which was tightly packed into polypropylene cylindrical vials (12 mm diameter). Vials were dried at ambient conditions for 48 h and then under vacuum for 24 h. Afterwards vials were cut into slices of 3 mm thickness and the slices were extensively washed in UHQ-water to remove sodium chloride. Subsequently, the samples were dried at ambient conditions for 48 h and then under vacuum for at least 24 h [22].

#### Material Surface Changes in Simulated Body Fluid (SBF)

In vitro simulated body fluid (SBF) test was applied according to the method proposed by Kokubo et al. [23]. SBF was prepared by dissolving the following chemicals (POCh, Poland) in UHQ-water:141 mM NaCl, 4 mM KCl, 0.5 mM MgSO_4_, 1 mM MgCl_2_, 4.2 mM NaHCO_3_, 2.5 mM CaCl_2_, and 1.0 mM KH_2_PO_4_. The resulting SBF was buffered to pH 7.40 with Tris(hydroxymethyl)aminomethane/HCl. The composite scaffolds were immersed in SBF solution in separate polypropylene containers with sample weight to SBF volume ratio 1:100 [g/ml^3^] and the samples were incubated at 37 °C for 7 and 14 days. Afterwards, the samples were washed in ethyl alcohol, air and vacuum dried to a constant weight. Microstructure of obtained composite scaffolds, their chemical composition before and after the SBF incubation were determined using scanning electron microscopy (NovaNanoSEM 200 FEI Europe Company, accelerating voltage 15 or 18 kV) coupled with energy dispersion X-ray (EDS) analyser. The EDS spectra were averaged for the whole analysed surface. Materials were analysed after coating with carbon.

#### Porosity Study

Effective porosity (η_e_) and differential pore size volume of the scaffold samples were determined by mercury intrusion porosimetry (PoreMaster60, Quantachrome Instruments, UK). The results of effective porosity measurements were performed in triplicates and they were expressed as mean and standard deviation (SD).

### Cell Culture Studies

All cell culture experiments on the studied bioactive scaffolds were performed in a standard growth medium without any additional osteogenic supplements.

#### Human BMSC Isolation and Culture Expansion

Unless stated otherwise, all cell culture reagents were purchased from Thermo Fisher Scientific. Human BMSCs were harvested from the iliac crest of adult patients (42–67 years old, both genders) according to the approved Institutional Review Board of the Jagiellonian University protocol (No. 1072.6120.254.2017). The mononuclear cell fraction was isolated using Ficoll-Paque (GE Healthcare), as described in the manufacturer’s protocol. Mononuclear cells were expanded in T-75 flasks (Nest Biotech) in a growth medium composed of alpha-Minimum Essential Medium (alpha-MEM), 10% mesenchymal stem cell qualified fetal bovine serum (MSC qualified FBS, Biological Industries) and 1% antibiotics (i.e. penicillin and streptomycin). Once the primary cultures reached 80–90% confluence, cells were detached from the bottom of tissue culture flasks with 0.25% Trypsin-ethylene-diamine-tetra-acetic acid (EDTA) and either used for experiments or further expanded in T-75 flasks. All experimental cultures were established with hBMSCs at passages 2–7.

#### Cell Seeding and Cultures on the Experimental Scaffolds

Before cell seeding, all types of SBG/PLGA experimental scaffolds each of the dimensions: 0.8–1 cm width; 0.5–0.6 cm height) were sterilized in 70% ethanol (water solution) for 30 min, washed five times with phosphate-buffered saline (PBS) to remove ethanol traces, exposed to UV light (30 min each side) and then left overnight in 6-well tissue culture plates under the laminar chamber to dry. HBMSCs were suspended at a density of 2 × 10^5^ cells per 100 μl of standard cell growth medium supplemented with 40 μl of fibrinogen (stock solution of 1.75 mg/ml PBS) and 2 μl of thrombin (stock solution of 25 U/ml in PBS) to initiate clot formations. Sterilized scaffolds were placed into separate wells of 24-well plates, and each scaffold was loaded with 140 μl aliquot of hBMSC suspension. Scaffolds were then incubated for one hour at 37 °C in a humidified 5% CO_2_ atmosphere to allow cells to attach to the scaffolds. Then, 2 ml of standard cell growth medium was carefully added per well. Following day 1, media were changed every 2–3 days, unless stated otherwise.

#### Ion Release Profiles from Cell-Loaded SBG/PLGA Scaffolds

Since our earlier work showed marked differences in the ion release profile of scaffolds with and without cells [24], we used cell-loaded scaffolds to examine the Ca, P and Si ions release to the culture medium. Briefly, the composite scaffolds loaded with human BMSC were inserted into separate wells of 24-well cell culture plates and immersed for up to 3 days in a growth medium. Culture media were exchanged every 24 h.Culture plates were incubated at 37 °C in a humidified, 5% CO_2_ atmosphere. Next Ca, Si and P ion release profiles were determined at 24, 48 and 72 h. The initial ion concentration in culture medium was assumed as a starting point and designated 0 h. For assessment of ion concentrations in the medium collected from hBMSC cells cultured on PLGA and SBG/PLGA composites, the inductively coupled plasma atomic emission spectrometry (ICP-OES, Plasma40, PerkinElmer, USA) was used. All tests were performed in triplicates and results were presented as mean ± standard deviation (SD).

#### Cells Visualization

To visualize cells on the studied scaffolds, hBMSCs were cultured for 3 days on experimental scaffolds. The scaffolds were then washed 3 times with PBS and fixed in 4% formalin in PBS for 20 min. Cell cytoplasm was stained with 5% eosin (Eosin Y, ethanol solution, Thermo Scientific) and cell nuclei with DAPI diluted 1:1000 in PBS. The reactions were developed for 5 min at room temperature. The images were taken using the Zeiss Axiovert CFL50 microscope with the use of fluorescent filters.

#### Migration Studies

Given the substantial ion release from the cell-loaded scaffolds at the first 3 culture days, they were incubated for 3 days in a growth medium and then the condition media (CM) were harvested for hBMSC migration studies. The latter were performed using colorimetric QCM Chemotaxis Cell Migration Assay (Sigma-Aldrich). Before cell seeding into the chambers (24-well plates), they were cultured in a serum free medium for 24 h in order to inhibit cell proliferation. HBMSC were then seeded in the upper chamber (8 μm pore diameter) at the density of 1.0 × 10^6^ cells/mL and exposed to condition media (CM) that had been harvested from the studied scaffolds and transferred to the lower chambers. Cells were incubated for 24 h at 37 °C. The control in this experiment was the growth medium that had been used to incubate the scaffolds for 3 days, but without any contact with the scaffolds. Cells that had migrated through the membranes were stained according to the manufacturer’s protocol, and the number of migrated cells was determined quantitatively by reading absorbance at 650 nm.

#### Effect of Conditioned Media (CM) on BMP-Responsive Reporter Mouse Osteoblast Cell Line BRITER

The effect of the above-mentioned CM harvested from hBMSC-loaded scaffolds on the BRITER mouse osteoblasts, the BMP-responsive reporter osteoblast cell line (Kerafast EBI001) was investigated as follows: Briefly, in this cell line, the reporter construct contains BMP responsive element (BRE) driven Firefly Luciferase gene (FFLuc) and SV40 promoter/enhancer driven Renilla luciferase (RRLuc) gene [25]. The latter serves as an internal normalization control for cell number as well as non-specific transcription activation. Upon BMP stimulation, cells increase Firefly Luciferase activity in a robust and sensitive manner. BRITTER cells were seeded at the density of 2 × 10^4^ cells/cm^2^ on 24-well plates and incubated for 24 h in a standard growth medium. Then the growth media were replaced by CM harvested from the cell-loaded scaffolds and Luciferase activity in BRITER cells was assessed after 3 h. The measurement of Luciferase activity was performed using dual luciferase assay (Promega).

#### Western Blot Analyses

Activation of signalling pathways in hBMSC cultured on the studied scaffolds was evaluated at day 3 culture. The scaffolds were washed 3 times in PBS, cut in halves, placed into 2-ml centrifuge tubes, covered with 300 μl of whole cell lysis buffer (Cell Signaling Technology), mixed and vortexed for 30 s to make sure that the buffer reached the inside of the scaffold. Then, the obtained cell extracts were transferred to new sterile 2 ml centrifuge tubes. Protein concentration in the extracts was determined with the MicroBCA kit (Pierce). Equal amounts of protein samples were separated on NuPAGE 4–12% Bis-Tris gels under reducing conditions, transferred to polyvinylidene fluoride (PVDF) membranes and then probed overnight at 4 °C with the following primary antibodies: anti-human Smad and phospho-Smad 1/5/9 antibodies (Cell Signaling Technology, #6944 and #13820, respectively), anti-human Tak1 and phospho-Tak1 (Cell Signaling Technology, #4505 and #9339, respectively), anti-human ERK1/2 and phospho-ERK 1/2 (Cell Signaling Technology, #9102 and #9101, respectively), anti-human p38 and phospho-p38 (Cell Signaling Technology, #8690 and #4511, respectively), and anti-human JNK and phospho-JNK (Cell Signaling Technology, #9252 and #4668, respectively). The horseradish peroxidase-linked secondary antibodies (GE Healthcare) were then applied for 1 h at room temperature and the peroxidase-based signal was detected using Western Lightning Chemiluminescence Reagent Plus (GE Healthcare). The signal was captured on Hyperfilm ECL chemiluminescent films (Perkin-Elmer).

#### Real-Time PCR Analyzes

Gene expression analyses were performed at hBMSC culture days 3, 7 and 21. Briefly, total RNA was isolated using TriReagent (MRC Inc.) and 0.5 μg of RNA from each culture was reverse-transcribed to complementary DNA (cDNA) with High-Capacity cDNA Reverse Transcription Kit (Applied Biosystems). The cDNA samples were used for real-time PCR analyses with TaqMan probes for Runx-2 (Hs01047973) and Osx (Hs01866874), cFos (Hs04194186) and Cx43 (Hs00748445). The reaction mixtures (15 μl total volume) for quantitative PCR (qPCR) consisted of 50 ng cDNA, 7.5 μl TaqMan Universal PCR Master Mix and 0.75 μl TaqMan probe. For PCR reaction with primers instead of probes, each reaction mixture (10 μL total volume) contained 50 ng cDNA, 1 μl of 0,5 μM gene-specific primers (Table 2), SYBR®Green I, AmpliTaq Gold® DNA Polymerase and the reaction buffer as recommended by the manufacturer (SYBR® Green PCR Master Mix, Applied Biosystems). All PCR reactions were carried out in a StepOnePlus Real-Time PCR thermal cycler (Applied Biosystems). The PCR reactions for TaqMan probe were performed for 40 cycles with denaturation step at 95 °C for 15 s, annealing at 60 °C for 1 min, and elongation at 60 °C for 1 min. For gene-specific primers reactions were performed for 40 cycles with denaturation step at 95 °C for 30s, annealing at 60 °C for 1 min, and elongation at 72 °C for 30s. Relative quantification (i.e. ddCT method) was used to analyse the results with cells cultured on PLGA as a reference.

**Table 2:** Oligonucleotides used in real-time PCR analyses

**Gene name**	**Forward (5′-3′)**	**Reverse (5′-3′)**
Col1	GTCTAGACATGTTCAGCTTTGTGGA	CTTGGTCTCGTCACAGATCACGTCAT
OPN	TGGAAAGCGAGGAGTTGAATG	CATCCAGCTGACTCGTTTCATAA
ON	GACTACATCGGGCCTTGCAA	GGGAATTCGGTCAGCTCAGA
BSP	AACGAAGAAAGCGAAGCAGAA	TCTGCCTCTGTGCTGTTGGT
OC	AAGAGACCCAGGCGCTACCT	AACTCGTCACAGTCCGGATTG
BMP 2	TGCTAGTAACTTTTGGCCATGATG	GCGTTTCCGCTCTTTGTGTT
BMP 6	AGACCTTGGTTCACCTTATG	CATCCACAAGCTCTTACAAC
Tata box-binding protein (*TBP*)	GGAGCTGTGATGTGAAGTTTCCTA	CCAGGAAATAACTCTGGCTCATAAC

#### Alkaline Phosphatase Activity Measurement

Alkaline phosphatase activity (ALP) was assessed in hBMSC cultured on experimental scaffolds for 7 days. Cell were then washed with sterile PBS and the culture medium from individual wells was replaced with 0.4 ml solution of 10% MTS reagent (CellTiter96Aqueous One Solution Cell Proliferation Assay; Promega) in phenol-free alpha-MEM. The development of colorimetric reaction was captured by measuring the absorbance at 490 nm. Afterwards, scaffolds were washed 3 times with PBS and ALP activity was measured kinetically as originally described by Osyczka and Leboy [26]. Briefly, protein extracts from the scaffolds were prepared with 300 μl of cell digestion buffer containing 1.5 M Tris (LabEmpire cat no. TRS001.1), 1 mM ZnCl_2_, 1 mM MgCl_2_ 6 H_2_O and 1% Triton X-100 (LabEmpire cat no.TRX777.100). The samples of 20 μl protein extracts were used for reactions with the 180 μl of cell assay buffer containing 1.5 M Tris (LabEmpire cat no. TRS001.1), 1 mM ZnCl_2_, 1 mM MgCl_2_ 6H_2_O and 1855 mg/ml of ALP substrate p-nitrophenol phosphate (pNPP) (SigmaAldrich). The changes in the absorbance at 405 nm were measured at 1 min intervals for up to 45 min. ALP activity was expressed as nmolpNPP/min/total volume of the protein extract and normalized to the number of viable cells estimated from the MTS assay.

#### In Vivo Ectopic Bone Formation and Histology

Verification of selected scaffolds was done in a rabbit model, basically as previously described [27]. This study was approved by the local Ethical Committee of the Jagiellonian University, decision no. 30/2020. The study was performed on 8 New Zealand white rabbits, 9–12 months in age and 3–4 kg weight. Before the implantation into *latissimus dorsi* muscles, the scaffolds were sterilized for 2 h in 70% ethanol under UV light, washed with PBS and then thoroughly dried under UV light at 40 °C. Each animal received 2 A1/PLGA and 2 S1/PLGA and 1 PLGA scaffold (each at the dimensions: 0.8–1 cm width; 0.5–0.6 cm height). In addition, sham surgery control (no scaffold) was included per each animal to assess the influence of the muscle pouch formation procedure itself. At 24 weeks post-surgery the animals were euthanatized, the implanted and sham control tissues with a margin of 2.5 cm of the muscle tissue were removed and preserved with 10% neutral buffered formalin pH 7,4 (Sigma). The 5 mm thick cross sections of fixed samples were prepared and stained for hematoxylin and eosin (H&E) (Hematoxylin 3G Tissue-Tek, Sakura, Japan, Eosin Tissue-Tek, Sakura, Japan) on a stainer (Tissue-TekPrisma, Sakura, Japan). Slides were evaluated under Olympus BX53 microscope and images and measurements were captured with digital image acquisition software (cellSens Dimention, Olympus, Poland). Histopathological evaluation focused on the detection of ossification. For morphometry, three representative images of areas containing apparent osteoid were captured at 200x magnification for each biomaterial group. Areas of osteoid were measured in μm^2^ for each image and ratio of osteoid surface to field surface was calculated.

### Statistical Analyses

All biological data were collected in triplicates and expressed as mean ± SD. Statistical analyses were performed in Statistica 13 (StatSoft®, USA) software. One-way analyses of variance ANOVA with *post-hoc* Tukey’s test was applied to calculate statistically significant differences at p < 0.05.

## Results

### Material Surface Changes in Simulated Body Fluid (SBF)

**FIG. 1:**
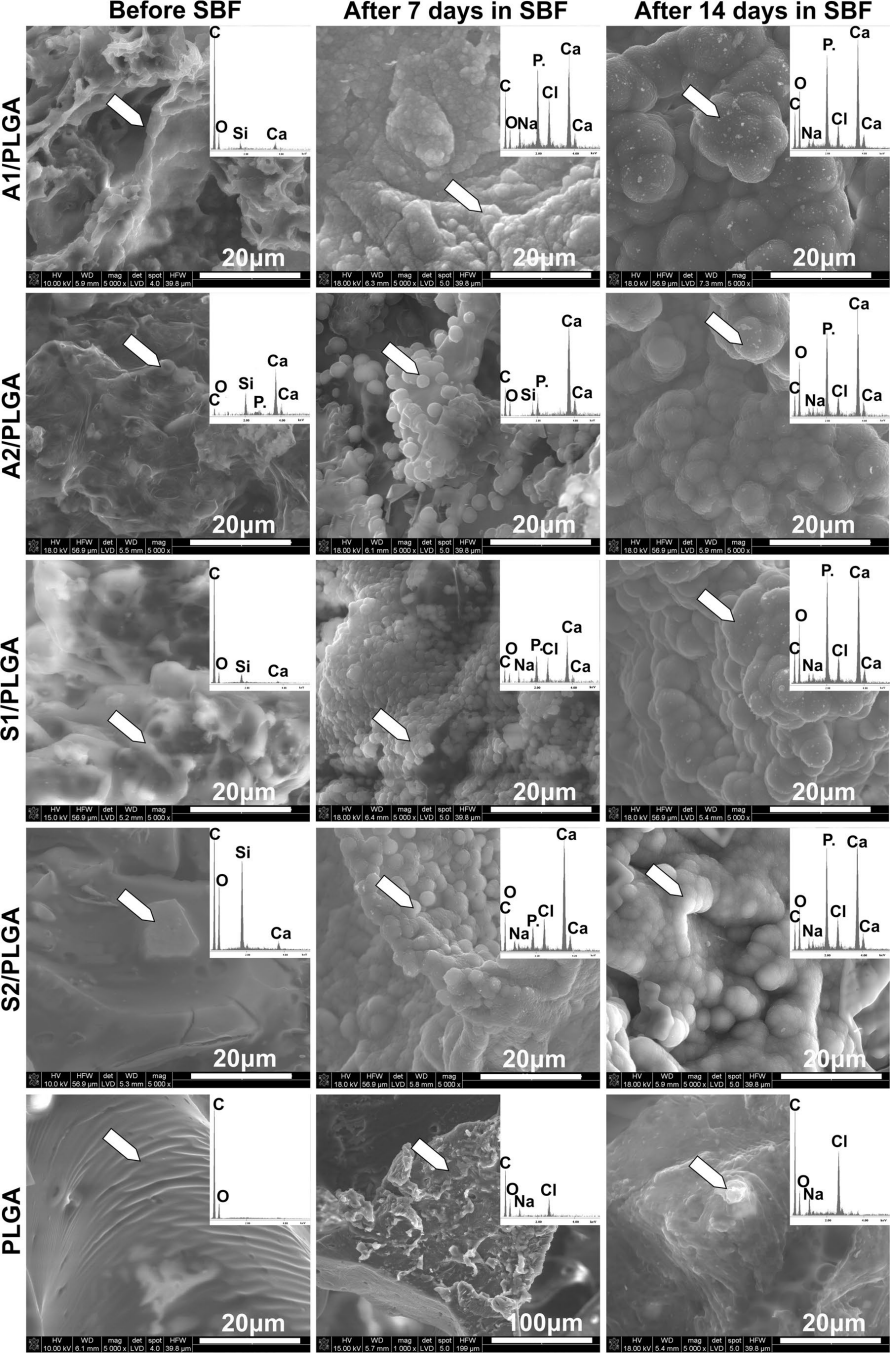
Surface morphology (SEM) and chemical composition (EDS) changes of the SBG/PLGA scaffolds in simulated body fluid (SBF) after 7 and 14 days of incubation

Calcium phosphate-forming ability of the scaffolds during the 7- and 14-day incubation in SBF is presented in Fig. 1. SEM images and EDS spectra of the materials were collected before and after SBF incubation. SEM images depicting composite scaffolds before SBF incubation showed that SBG particles were homogeneously distributed in the PLGA polymer matrix and consequently this also increased micro-roughness of the composites surfaces. The PLGA scaffold surface was the smoothest with few irregularities. During incubation in SBF, PLGA scaffold did not show any chemical and morphological changes related to calcium phosphates forming, at any given time of analysis. Trace amounts of Na and Cl were observed by EDS analyses for all studied scaffolds, including PLGA, most probably due to trace precipitation of NaCl from the SBF solution. After 7 days of SBF incubation all studied composite scaffolds were covered with spherical cauliflower-like crystals rich in Ca and characteristic of carbonated hydroxyapatite (HCA). These crystals were aggregated and partially linked but did not form continuous layers. After 14 days of incubation the size of depositions increased in all studied composite scaffolds and they formed an uniform layer on the scaffold surface and inside the scaffolds pores. Moreover, higher concentrations of Ca and P were detected after 14 days of SBF immersion of the composites. A lack of Si in the EDS spectra after 7 and 14 days of SBF incubation of the composites suggests that the surface layers were thick, successfully reducing Si ions exchange (right after 72 h of incubation, reported by ICP-OES (Fig. 3c)), and probably partially reducing the effects of the potential siloxane groups exposition. Overall, the results indicated that the chemical composition of SBG in the PLGA-based composites did not affect the formation of calcium phosphate (CaP) layer, its kinetics or morphology.

### Porosity

**FIG. 2:**
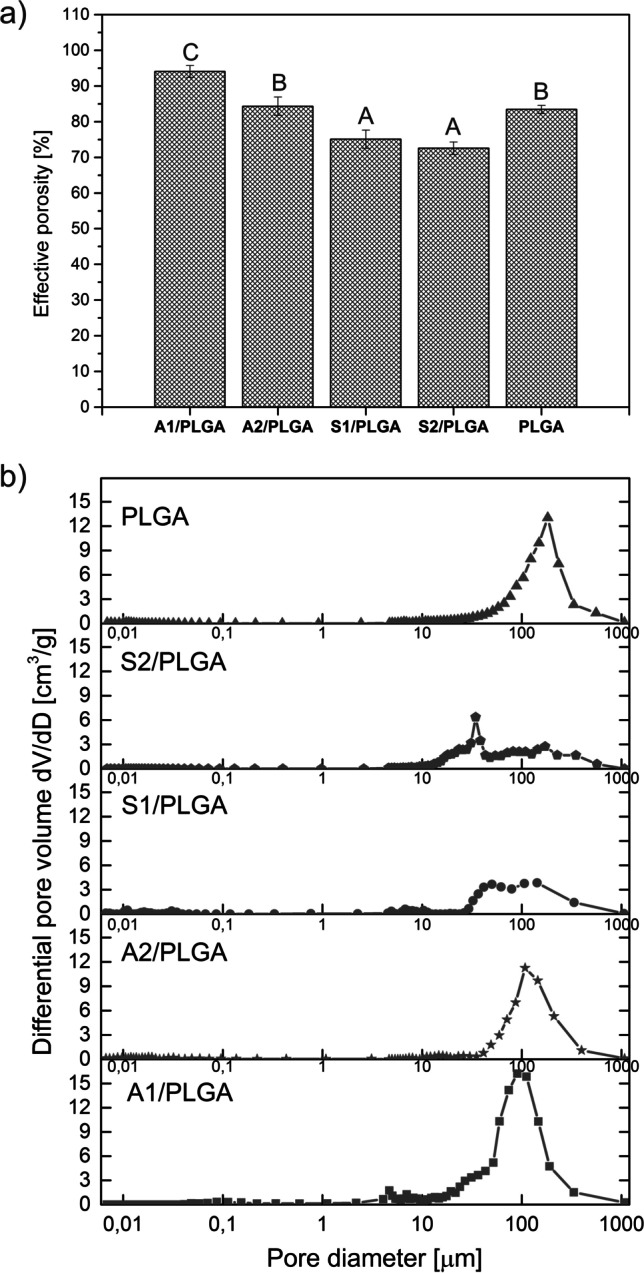
Effective porosity (a) and differential pore volume distribution (b) of SBG/PLGA scaffolds. Statistically significant differences (p < 0.05) in the effective porosity between the different scaffolds types are indicated by different upper case letters. Bars marked with the same letters are not statistically different

The effective porosity and differential pore volume of the obtained scaffolds are presented in Fig. 2. The highest effective porosity (Fig. 2a) was detected for the A1/PLGA scaffolds (94%). A2/PLGA and PLGA scaffolds displayed lower porosities of 84.3% and 83.4%, respectively. The lowest porosities were found for the scaffolds obtained with SBGs type S, i.e. S1/PLGA - 75% and S2/PLGA – 72.5%. Thus, it could be concluded that composite scaffolds containing type A SBGs exhibited higher effective porosity values compared to the ones containing SBGs type S. Moreover, the scaffolds obtained with binary system SBGs showed higher porosities vs. the ones obtained with the ternary system SBGs, although that was significant only for A type SBGs. Furthermore, for A1/PLGA scaffolds three main pore volume fractions could be distinguished (Fig. 2b): small fraction with the diameters in the range of 5–10 μm, medium fraction with diameters of approximately 50 μm, and a large, prevalent pore fraction of the diameters of c.a.100 μm. For A2/PLGA material a more uniform pore size distribution was registered with the large pores of the diameter c.a.100 μm. S1/PLGA material exhibited three pore volume fractions including small, medium and large pores. Interestingly, large pore fraction was the dominant one in S1/PLGA as in A2/PLGA and A1/PLGA. In contrast, for S2/PLGA scaffolds bimodal pore fractions were detected (i.e. medium and large), but the dominant one was the medium fraction. PLGA scaffold showed only a large pore fraction.

### Ion Release from Cell-Loaded Scaffolds

**FIG. 3:**
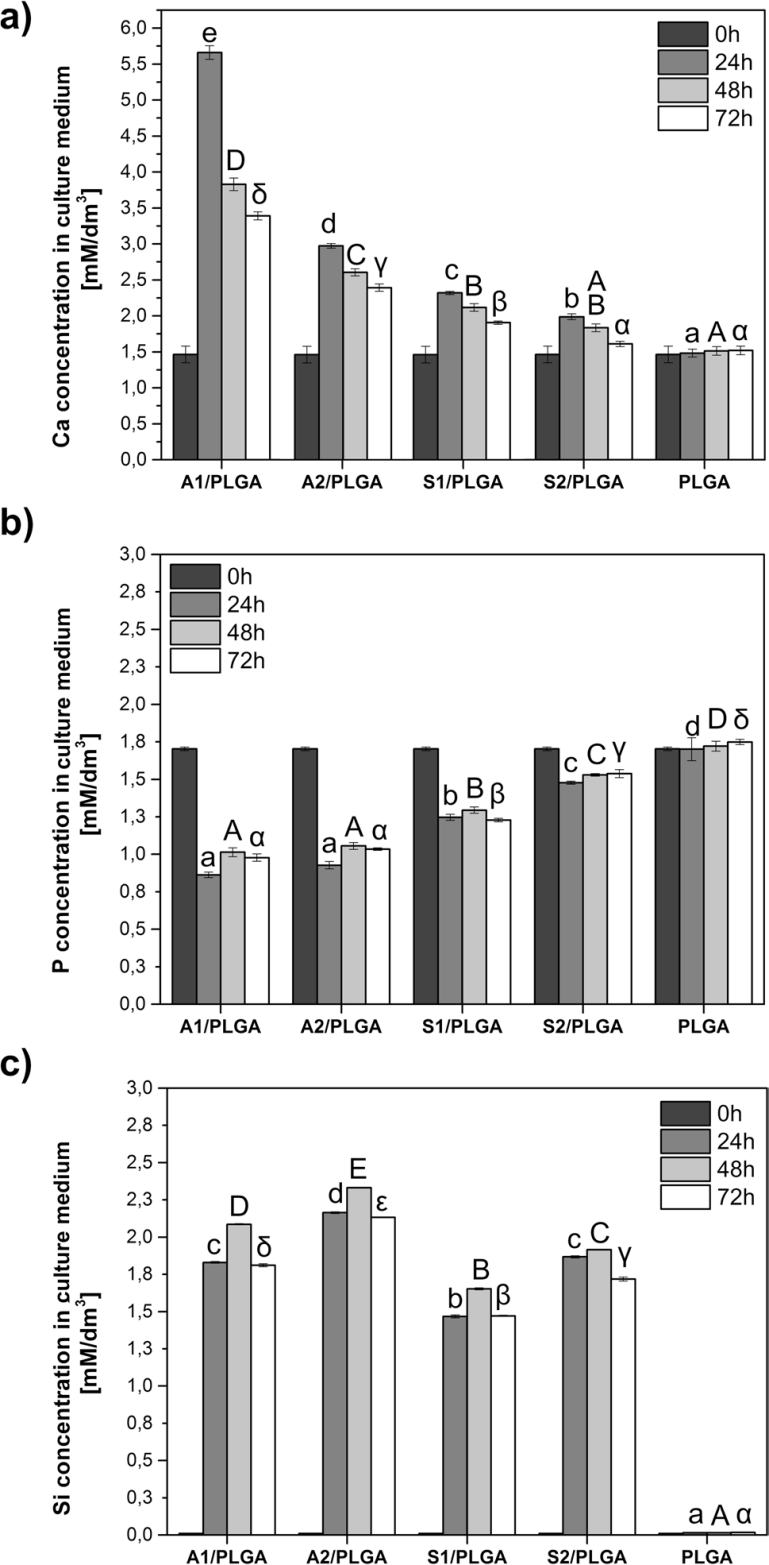
Ca (a), P (b), and Si (c) concentrations (ICP-OES) in the culture medium after incubation of hBMSC-loaded SBG/PLGA scaffolds for up to 72 h. Day 0 stands for the ion composition of plain culture medium. Statistically significant differences (p < 0.05) in the ions concentration either within a scaffolds group at different soaking periods or between different scaffolds types at indicated soaking period are marked by different upper and lower case Arabic and Greek letters. Bars marked with the same letters are not statistically different

Ion release profiles from the scaffolds loaded with human BMSC and incubated for up to 3 days in a growth medium, are presented in Fig. 3. All studied SBG/PLGA composite scaffolds except control PLGA, exhibited a common pattern of Ca ions exchange (Fig. 3a). Namely, the highest Ca concentrations in culture media were observed at 24 h incubation and then their levels decreased gradually after 48 h and 72 h. Ca concentration in the culture medium at each studied incubation time point was statistically higher than the initial Ca concentration (1.46 mM/dm^3^) – i.e. upon immersion of scaffolds (time 0). Regarding Ca release, materials could be arranged in the following order from the highest to lowest Ca release to the culture medium: A1/PLGA > A2/PLGA > S1/PLGA > S2/PLGA. The highest amounts of Ca (5.66 mM/dm^3^) were released from A1/PLGA after 24 h incubation. Notably, PLGA as a control material did not exchange Ca, P, nor Si ions with the incubation medium, and thus at any studied time Ca levels stayed relatively constant and without any statistical differences between different incubation time points. Given the above, the amounts of Ca released to culture media correlated to the Ca content in the SBGs and to their CaO/SiO_2_ ratio. Notably, Ca exchange rate for the materials containing ternary system SBGs was lower than for their analogues containing binary system SBGs. As it was discussed in our previous study [24], lower Ca dissolution from the SiO_2_–CaO–P_2_O_5_ SBGs is connected with the phosphorus presence, as the latter contributes to the phase separation in the structure and creates the calcium-phosphate rich clusters. Moreover, calcium silicate crystallization and thus the stabilization of silicate network limit ternary materials dissolution and this is noticeable especially in Ca dissolution process [20]. Changes of the P concentration in culture media over the first 3 incubation days had similar pattern for all studied SBG/PLGA scaffolds (Fig. 3b). This included a decrease in phosphate concentrations over whole incubation period, with the most rapid decrease during the first 24 h of incubation. The highest phosphate adsorption was recorded for A1/PLGA and A2/PLGA scaffolds and the lowest for S2/PLGA. Thus, as for phosphate adsorption, the scaffolds could be arranged as follows: A1/PLGA = A2/PLGA < S1/PLGA < S2/PLGA. We assume the decrease in P concentration in culture medium may either indicate its adsorption to the materials surface or it may be associated with cellular-based processes. Finally, the Si was released from all studied scaffolds at the similar levels and rate and the highest Si amounts in the culture medium were detected 24–48 h (Fig. 3c). In total, the highest Si levels were recorded for A2/PLGA scaffold and the lowest for the S1/PLGA, giving the overall profile for the studied scaffolds: A2/PLGA > A1/PLGA> S2/PLGA > S1/PLGA. Thus, despite higher content of Si in the S type composite scaffolds, their surface activity was overall lower that the type A scaffolds resulting in lower release of Si ions. This could be also a result of the different porosity of the scaffolds, because greater porosity of type A scaffolds could contribute to improved ion release from glass particles.

### Human BMSC Analyses

**FIG. 4:**
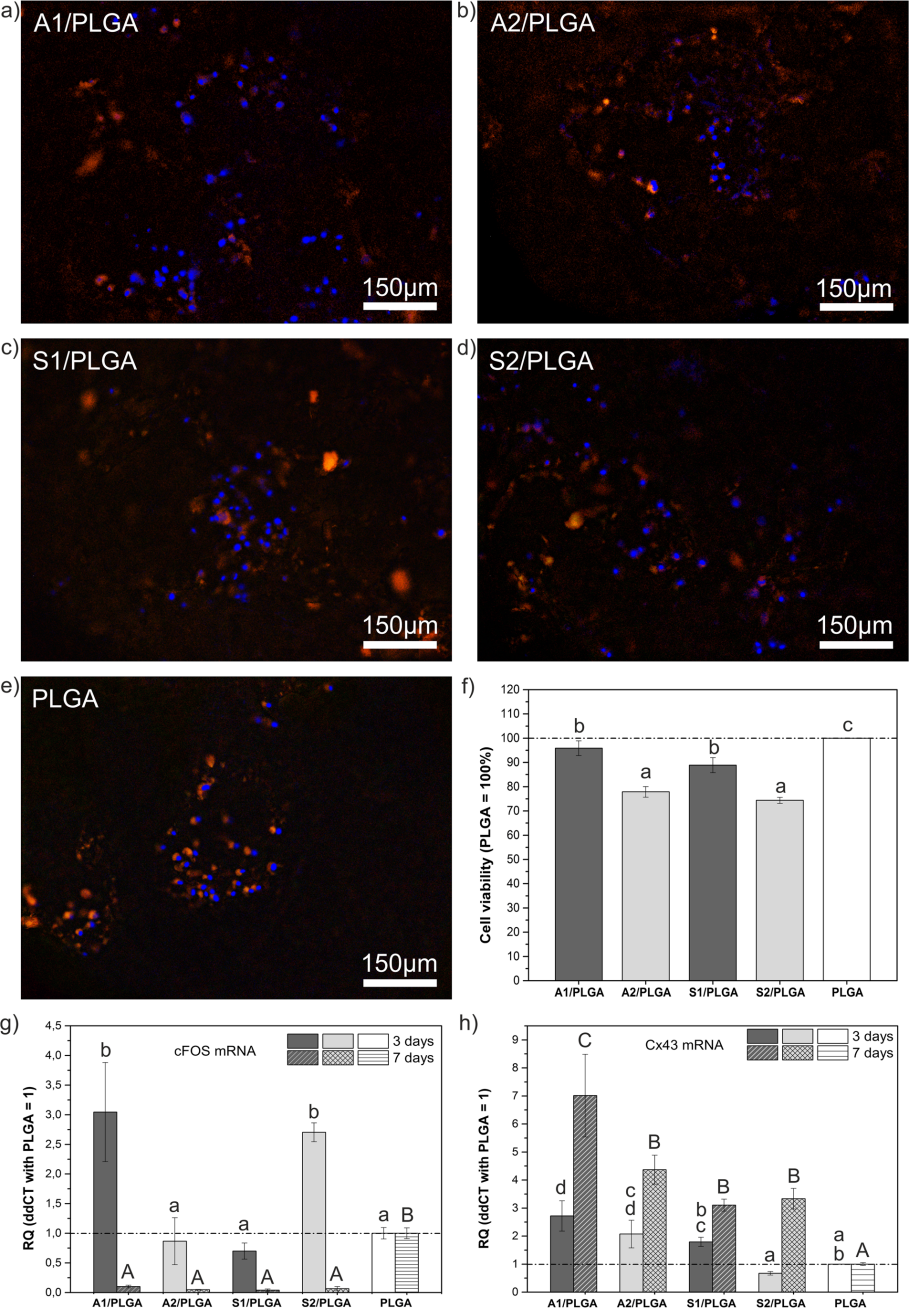
Distribution of hBMSC on the studied SBG/PLGA scaffolds (a-e) at culture day 3. Cells cytoplasm is stained with eosin (orange) and nuclei with DAPI (blue); cells viability (colorimetric assay) at culture day 7 (f); cFOS (g) and Cx43 (h) mRNA expression levels at days 3 and 7 cultures. Quantitative values obtained for PLGA scaffolds (reference) are marked with a dash line. Statistical significant differences (p < 0.05) between different scaffold types at given time point are indicated by different lower or upper case letters. Bars marked with the same letters are not statistically different

Imaging of human BMSC grown on the studied scaffolds showed that the scaffolds were suitable for their colonization by hBMSC (Fig.4). Cells were well distributed within the scaffolds and we have not observed any obvious differences in the cells distribution depending on the scaffolds type (Fig.4a-e). Though, quantitative data regarding live cell analysis at day 7 culture (Fig. 4f) showed lower numbers of live cells in group 2 composites (i.e. the ones containing ternary SBGs) vs. respective group 1 composite scaffolds. Moreover, at day 7 culture all scaffolds except for A1/PLGA showed lower numbers of live cells vs. PLGA. To explore further the potential cell-material and cell-cell interactions, we have also evaluated cFos and Cx43 mRNA expression levels (Fig. 4 g, h) in cultures grown for 3 and 7 days on the composite scaffolds. In general, expression of Cx43 increased from day 3 to 7 on all studied scaffolds and at day 7 it was significantly higher than that on PLGA control. It is thus plausible that the chemical compositions of SBG/PLGA scaffolds facilitated cell-cell communication. Furthermore, Cx43 expression was the highest for cells grown on A1/PLGA scaffolds and decreased with decreased CaO/SiO_2_ ratio, indicating partial dependence of Cx43 expression on the calcium content in particular scaffolds. In contrast, cFos expression was high at day 3 on A1/PLGA and S2/PLGA and decreased beyond PLGA control levels on all studied composite scaffolds at day 7. Stimulation of cFos can reflect cells response to calcium ions [28]. Nonetheless, in the present study, we observed increased cFos expression for both high- and low-calcium scaffolds and this was independent of the ion release to the culture medium.

**FIG. 5:**
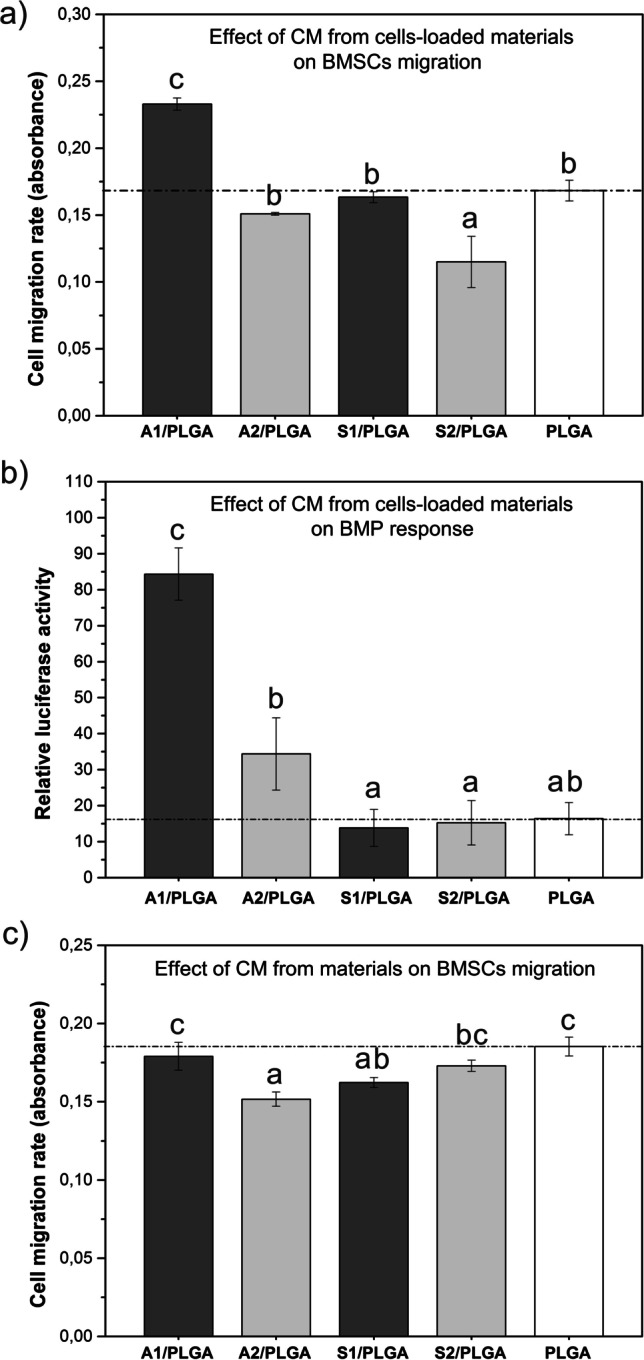
The effects of condition media (CMs) collected from the hBMSC-loaded scaffolds on hBMSC migration rate (a), Luciferase activity in BMP-responsive mouse osteoblasts reporter cell line (BRITE) (b), and the effect of condition media (CM) collected from the culture medium incubated scaffolds on hBMSC migration rate (c). Quantitative values obtained for PLGA scaffolds (reference) are marked with a dash line. Statistical significant differences (p < 0.05) between different scaffold types are marked by different letters. Bars marked with the same letters are not statistically different

Cell migration studies revealed that CM collected from cell-loaded A1/PLGA scaffolds increased the rate of hBMSC migration (Fig. 5a). It should be noted that CM collected from empty scaffolds did not have any effect on cell migration (Fig. 5c.), suggesting that the “conditioning” of the cells on the A1/PLGA scaffolds was necessary for hBMSC to produce signalling factors that stimulated cell migration.

**FIG. 6:**
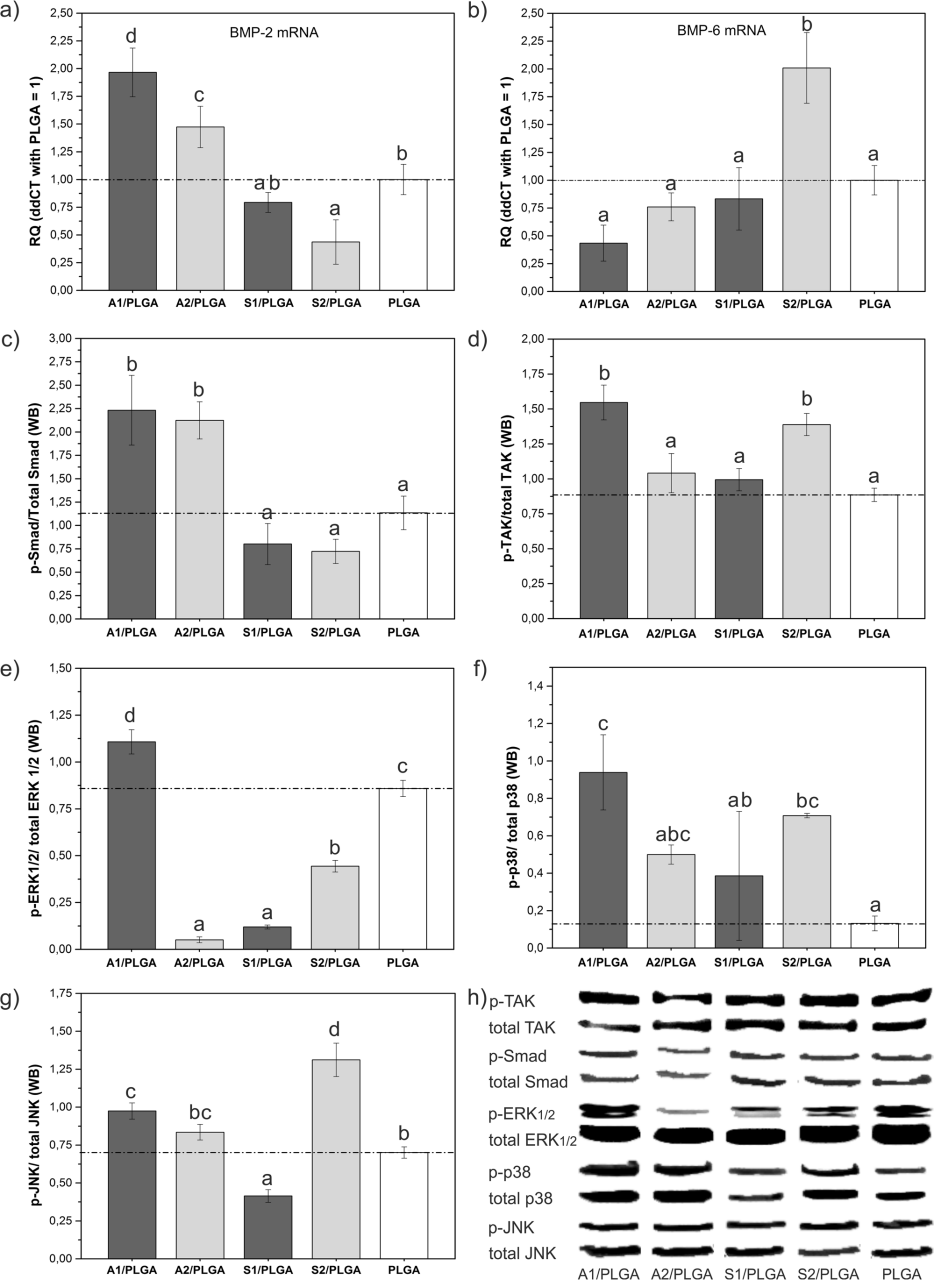
Expression levels of BMP-2 (a) and BMP-6 (b)mRNAs in hBMSC cultured for 3 days on composite scaffolds and the activation of BMP-related signaling pathways at day 3 cultures: Densitometry analyses of Western blot results for phospho-Smad1, 5, 8 (c), phospho-TAK1 (d); phospho-ERK1/2 (e), phospho-p38 (f) and phospho-JNK (g). The Western blot results are shown in (h). Quantitative values obtained for PLGA scaffolds (reference) are marked with a dash line. Statistical significant differences (p < 0.05) between different scaffold types are marked by different letters. Bars marked with the same letters are not statistically different

Furthermore, CM harvested from hBMSC-loaded A1/PLGA scaffolds was the only one that stimulated Firefly Luciferase in BRITER cells (Fig. 5b), thus on contact with A1/PLGA hBMSC may have produced substantial amounts of BMPs suitable to activate BMP-responsive reporter construct in BRITER cells. Indeed, hBMSC analyses at day 3 culture revealed significantly higher BMP-2 mRNA levels for cells grown on A1/PLGA and A2/PLGA scaffolds (Fig. 6a) and BMP-6 mRNA levels for cells grown on S2/PLGA scaffolds (Fig. 6b), compared to PLGA control. Subsequent analyses of intracellular signalling pathways activated by hBMSC grown for 3 days on the composite scaffolds showed that the cells were capable to activate both BMP canonical and non-canonical pathways, as the levels of phospho-Smad1, 5, 8 were increased for A1/PLGA and A2/PLGA (Fig. 6d) and phospho-Tak1 for A1/PLGA and S2/PLGA scaffolds (Fig. 6c-e).

Further analyses revealed markedly higher activation of ERK (Fig. 6e) in cells cultured on A1/PLGA, phospho-p38 in cells cultured on A1/PLGA, A2/PLGA and S2/PLGA (Fig. 6f) and phospho-JNK (Fig. 6g) in cells cultured on A1/PLGA and S2/PLGA vs. PLGA. Notably, P_2_O_5_ addition to high-calcium SBGs seemed inhibitory for all (except for Smad) analysed pathway, whereas addition of P_2_O_5_ stimulatory for high-silica ones.

Giving that overall activation of signalling pathways was the most profound on A1- and S2/PLGA composites, there is also possible cooperation between BMP signalling and cFos expression in hBMSC grown on the studied composite scaffolds (Fig. 4g) as it was suggested for some other cell types [29].

**FIG. 7:**
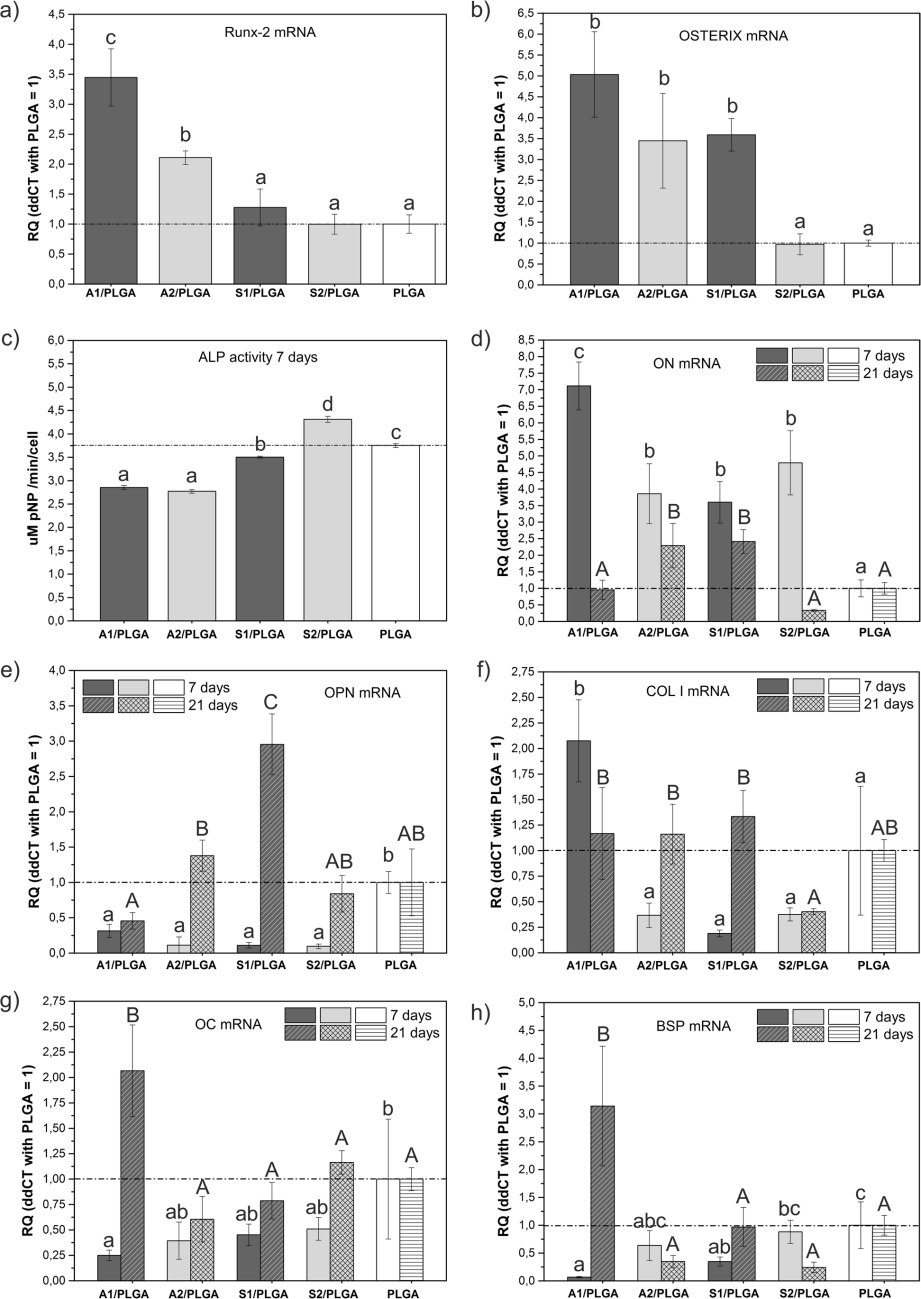
Expression levels of osteogenic transcription factors Runx-2 (a) and Osterix (b) mRNAs at day 3 hBMSC culture; ALP activity (c) at day 7 hBMSC culture and mRNA expression levels of bone matrix-related osteonectin (ON, d), osteopontin(OPN, e), collagen type I (COL I, f), osteocalcin (OC, g) and bone sialoprotein (BSP, h) at day 7 and 21 cultures. Quantitative values obtained for PLGA scaffolds (reference) are marked with a dash line. Statistical significant differences (p < 0.05) between different scaffold types at given time point are indicated by different lower or upper case letters. Bars marked with the same letters are not statistically different

Finally, the expression of osteogenic transcription factors Runx2 and Osx (Fig. 7a, b) could be detected as early as day 3 culture. The former was elevated for cells grown on A1/PLGA and A2/PLGA and decreased to control PLGA levels for S type scaffolds. The presence of P_2_O_5_ seemed inhibitory for type A scaffolds, consistent in part with our previous study [24] showing higher Runx2 activation for thin films without P_2_O_5_. Osx expression was increased for all studied SBG/PLGA scaffolds except for S2/PLGA, but the latter scaffolds were the only ones to increase ALP activity of hBMSC at day 7 (Fig. 7c). Further gene expression analyses showed the highest mRNA levels of ON, OPN, Col I, OC and BSP (Fig. 7d-h) for cells cultured on A1/PLGA. Thus, it was plausible to assume that these scaffolds represent the most potent ones to induce and maintain osteogenesis of hBMSC in long-term cultures without the requirement for addition of osteogenic growth factors to the culture medium to support osteogenesis.

### In Vivo Verification of Selected Scaffolds

**FIG. 8:**
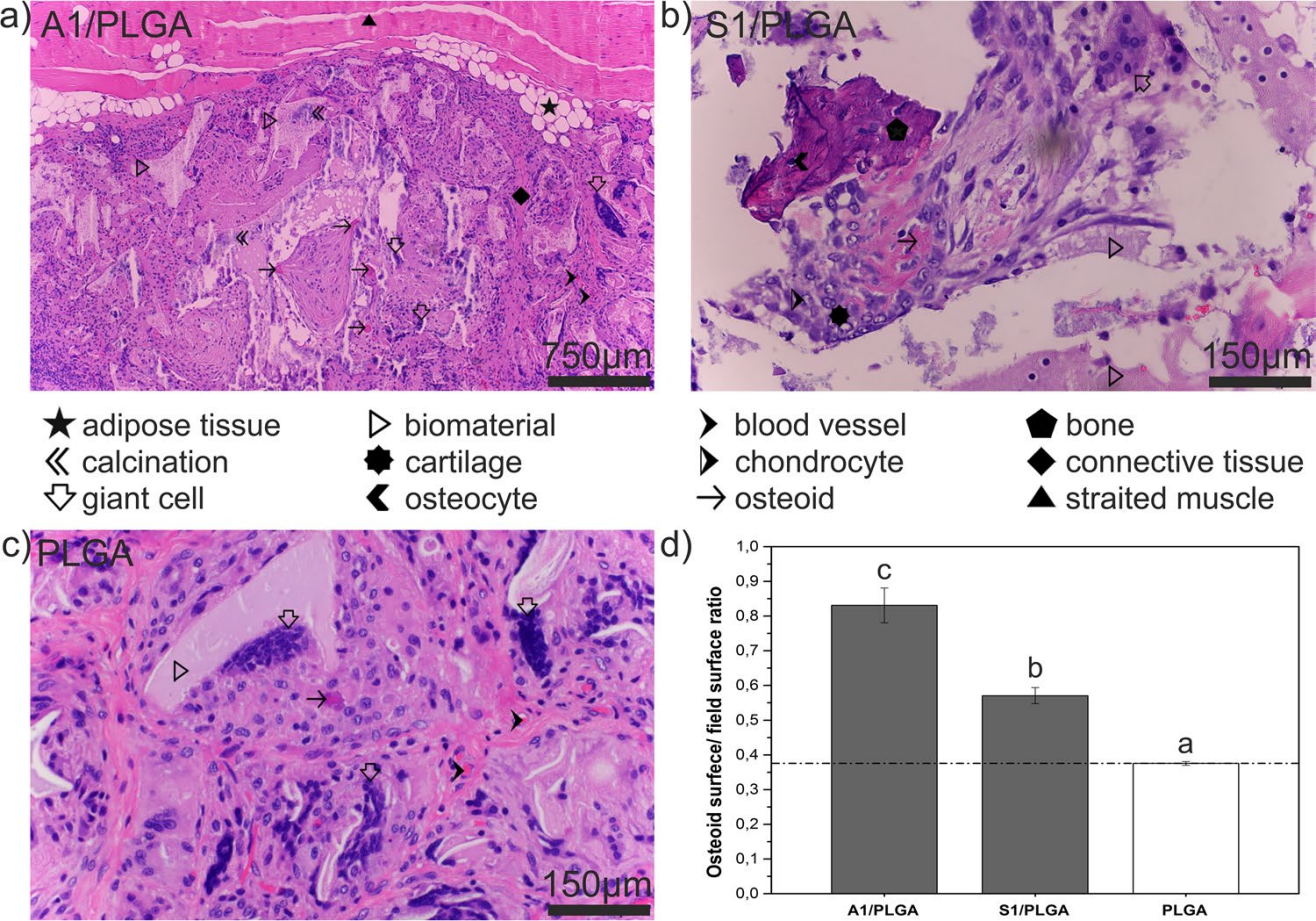
Hematoxylin/eosin staining of tissue sections obtained 24-weeks post-implantation of A1/PLGA (a), S1/PLGA (b) and PLGA (c) into *latissimus dorsi* muscles of New Zealand white rabbits and morphometry of osteoid in the representative tissue sections (d). Quantitative values obtained for PLGA scaffolds (reference) are marked with a dash line. Statistical significant differences (p < 0.05) between different scaffold types are marked by different letters

Giving that A1/PLGA scaffolds appeared the most promising osteoinductive constructs, we subjected A1/PLGA and S1/PLGA (i.e. the constructs containing binary SBG) along with PLGA control to a classic “ectopic bone formation” [30]. H/E staining of obtained tissue sections (Fig. 8) revealed partial degradation of all types of implanted scaffolds and their replacement by connective tissue containing abundant giant cells. Connective tissue varied from pauci cellular with rich collagenous stroma to highly cellular spindle cell areas. Formation of new blood vessels was present and in some cases focal lymphocytic inflammatory infiltrates or areas of adipose tissue were also observed, but with no obvious differences for different scaffold type. In all reviewed cases distinct foci of osteoid were found. For S1/PLGA scaffolds (Fig. 8b) bone trabeculae were also spotted and in one case additionally small foci of cartilage were present, some of them undergoing ossification. A1/PLGA scaffolds (Fig. 8a) exhibited similar pattern of changes and did not differ significantly among themselves. On the other hand, for S1/PLGA scaffolds tissue responses were less uniform, with some sections showing only a small amount of osteoid to sections displaying bone and cartilage formation. Morphometric analysis (Fig. 8d) focused on the measurement of amount of osteoid by arbitrarily defined parameter: mean osteoid surface/field surface ratio, measured under 200x magnification. With this approach, the largest amount of osteoid was found for A1/PLGA scaffolds and the least amount was present in PLGA group (Fig. 8c).

## Discussion

In this work we explored further the idea that the endogenous BMP expression and activation of BMP-related pathways in hBMSC can be a key molecular signature of osteoinductive materials. Previously we showed that PLGA-based composites enriched with gel-derived bioactive glasses of either SiO_2_–CaO or SiO_2_–CaO–P_2_O_5_ systems were capable to induce BMP signalling and prompt osteogenesis of human BMSC when prepared in the forms of thin films and used as growth surfaces. Such biological response was achieved without any additional osteogenic stimuli but cell response depended partly on the CaO/SiO_2_ ratio of bioactive glass and its P_2_O_5_ content and only partly relied on the ions released from the material surface [24]. We thus asked whether the osteoinductive potential of such materials is maintained upon 3D architecture and to what extent it relies on ions released from 3D structures. To address this, we chose two SBG/PLGA composites of different CaO/SiO_2_ ratio, both with and without P_2_O_5_ to examine in more details their potential to induce and maintain osteogenesis of hBMSC when prepared in the form of 3D porous scaffolds. As with our previous study [24], no other osteogenic stimuli were used besides the materials.

All studied SBG/PLGA composite scaffolds were bioactive as verified by SBF test and formed porous 3D structure. Regarding the latter, there were important differences in the effective porosity and pore volume distribution depending on the scaffold type, that could have contributed to ion exchange rate of the material surface as well as to cell response. Effective porosity was the highest for A1/PLGA scaffolds and these scaffolds released also the highest amounts of calcium ions in the first 24 h of incubation in the culture medium. Addition of P_2_O_5_ to the SBG composition decreased A2/PLGA scaffolds porosity and Ca ions exchange rate, but still type A composite scaffolds were more porous vs. type S scaffolds, displaying a prevalent pore size fraction of about 100 μm. In the binary SiO_2_–CaO system, calcium oxide was found to depolymerize silicate structure, while in the ternary glasses the Ca^2+^ partition between the silicate part of the glass structure and the phosphate part suggest the increasing network connectivity in silica matrix, and thus enhanced chemical durability. The presence of orthophosphate/diphosphate groups charge-balanced by modifier cations Ca^2+^ results in decreased calcium release compared to binary system without P_2_O_5_. Overall, surface activity of type S scaffolds was lower in terms of ions exchange rate and these scaffolds were less porous, with the most significant changes for S2/PLGA, where the medium sizes pores about 50 μm were the dominant fraction. The structure of high silica S type glasses is more rigid and polymerized versus A type materials, and thus the reduction in solubility of S type glasses was observed. Besides composition and structure, the solubility of bioactive glasses in the SBG/PLGA composites could have been affected by the porosity of the scaffolds. The higher porosity results in better surface accessibility and improved ion release from glass particles. Notably, the scaffolds containing type A glasses displayed the same pattern of Ca and P exchange as respective films [24], but released relatively less Ca and adsorbed less P. In contrast, Ca release and P adsorption was slower for type S scaffolds, and they also adsorbed relatively less P vs. films. Both scaffolds and previously studied films released Si with the same pattern, but the scaffolds released Si faster and higher amounts vs. films. In the case of composite films, the surface of bioglass particles is more accessible (due to sedimentation), which directly contributes to both a massive release of Ca^2+^ from SBG and chemisorption of ions (Ca^2+^, PO_4_^3−^) from the incubation solution/culture medium. This effect limits the release of Si ions due to the rapid formation of HAp layer, and in the case of cell culture, the uniform film surface promotes rapid cell adhesion which inhibits the dissolution of silica bond. Relatively lower dissolution of Ca^2+^ ions from 3D scaffolds, despite the larger surface area of interaction with the environment comes from the overall less exposed bioglass particles compared to the 2D forms. Furthermore, the lower supersaturation of the solution and slower surface changes promote further breaking of Si–O–Si bonds by the action of –OH groups and cause the dissolution of the glass network. This results in the release of soluble silica into the incubation solution. In addition, the adhesion of cells and organic molecules from the medium, due to the 3D structure, may not occur over the whole surface of composites. Thus, this indirect comparison of films [24] and scaffolds (this work) of the same composition suggests different surface activity of these materials, depending on the materials architecture.

Regardless of the chemical composition of SBG/PLGA architecture, we confirmed that the scaffolds were suitable to support the growth of hBMSC and promoted cell-cell interaction as represented by Cx43 expression. Whereas the expression of Cx43 may indicate cell-cell communication, the expression of cFos can be indicative of cell-biomaterial surface interactions [24, 31, 32]. Notably, at day 3 culture, cFos expression was the highest for A1- and S2/PLGA composite scaffolds, but its expression was negligible for all studied composites at day 7 culture. This may reflect the process of cell responses after seeding to the scaffold, where cell-biomaterial interactions are most important at the initial culture stage and they are replaced by cell-cell interactions at further culture stages. In this view, human BMSC would best interact with the A1/PLGA and S2/PLGA surfaces as revealed by cFos expression at day 3 culture, followed by increased cell-cell communication on these and other scaffolds, as revealed by Cx43 expression at day 7. Although increased cFos expression may also reflect an early cells response to calcium ions [33, 34], this is not a case for currently studied chemical compositions, as increased cFos expression was noted for both A1/PLGA and S2/PLGA, that differed markedly in the CaO/SiO_2_ ratio of SBGs as well as Ca release levels at the initial incubation stages in culture medium. It is also worth mentioning that, in the present study, the cells were seeded in a fibrin clot. This cell-seeding method determines different cell distribution on the 3D scaffolds (i.e. more spatial and closer to in vivo tissue complexities) compared to the uniform and flat cell distribution on 2D material. Moreover, during natural bone healing, broken fragment is exposed to the action of blood cells that also form blood clots at the site of bone damage [35, 36]. The latter also promotes the repair of damaged tissue. Therefore, the use of the fibrin-clot method of cell seeding on 3D scaffolds brings the processes taking place in the scaffolds closer to in vivo conditions vs 2D films where it is not necessary or even disadvantageous to use such method. Giving that the material surface activity and ion exchange rate with the physiological fluids may contribute to cell response, we investigated the potential of condition media (CM) harvested from hBMSC-loaded scaffolds to attract further human BMSC and activate BMP promoter in BRITER cells. We observed that human BMSC migration as well as luciferase activity of BRITER cells were significantly increased when the cells were exposed to CM collected from A1/PLGA scaffolds pre-cultured with BMSC (Fig. 5a, b). Notably, CM from empty scaffolds did not have any effects (Fig. 5c), which further supports the importance of studying the bioactive materials ion release profile and their biological action when they are loaded with cells rather than examining their surface activity as prepared. Considering that the A1/PLGA scaffolds released the highest levels of Ca and moderate levels of Si and they displayed the highest differential and cumulative pore volume, it is plausible to assume, that both the physical and chemical properties of these scaffolds contributed to hBMSC response and resulted in the release of chemotactic factors that consequently stimulated hBMSC migration and activated BMP response.

Bone morphogenetic proteins belong to chemotactic growth factors which can stimulate hBMSC migration [37] and the culture media collected from hBMSC loaded A1/PLGA scaffolds were the only ones that stimulated luciferase activity in BRITER cells [24]. In fact, we detected high BMP-2 mRNA levels in hBMSC grown on A1- and A2/PLGA and high levels of BMP-6mRNA in hBMSC grown on S2/PLGA scaffolds. Furthermore, we observed the activation of BMP-related SMADs and Tak1, which were significantly elevated for cells grown on A1/PLGA (both SMAD1, 5, 8 and Tak1) and S2/PLGA (Tak1 only) scaffolds. Notably, the expression of BMP types had different pattern, depending on chemical composition of composites and the high BMP-2 expression for cells grown on A2/PLGA scaffolds was not sufficient to induce both canonical and noncanonical BMP signalling. It is plausible that additional to BMP-2 and -6 BMP subsets could have contributed to the activation of the intracellular BMP signalling on A1- and S2/PLGA. Further exploration of Tak1- dependent signalling showed the activation of ERK on A1/PLGA scaffolds that corresponded well with high calcium release from these scaffolds. It has been documented that ERK can be activated by high extracellular calcium [38]. In this work, higher calcium levels were released to the culture medium during the first 3 culture days on A1/PLGA materials vs. A2/PLGA and ERK activation on A1/PLGA was significantly higher than on A2/PLGA. In contrast, cells grown on S2/PLGA activated p38 and JNK. It has been recently showed that silica nanoparticles can activate p38 pathway in BEAS-2B and HBEC3-KT cells [39], JNK pathway in human bronchial epithelial cells [40] and murine RAW 264.7 macrophage cell line [41]. These pathways could have contributed to some pro-inflammatory responses [42, 43] that prevented terminal differentiation of BMSC on our high-silica composites, as presented by low osteocalcin and bone sialoprotein levels. Furthermore, we observed higher levels of p-p38 and p-JNK in cells grown on S2/PLGA vs. S1/PLGA scaffolds that corresponded with higher silica release during first 3 culture days on S2/PLGA vs. S1/PLGA. Overall, it can be assumed that the presence of P_2_O_5_ decreases calcium release from A2/PLGA and increases silica release from S2/PLGA scaffolds, which is then reflected in opposite biological responses of high-calcium and high-silica materials with and without P_2_O_5_.

Examining early osteogenic transcription factors showed that the expression levels of Runx-2 and Osx were generally higher for cells cultured on the scaffolds without P_2_O_5_. This may indicate that the presence of P_2_O_5_ in the scaffolds slows down the induction of osteogenesis. Moreover, the expression of both Runx-2 and Osx was detected for high-calcium scaffolds only and in fact only cells grown on A1/PLGA scaffolds showed expression of late osteoblast markers such as osteocalcin and bone sialoprotein. This suggests that the induction of BMP signaling pathways together with the high expression of Runx2 and Osx is required to complete osteogenesis of cells cultured on SBG/PLGA composites. Similar to Osx expression pattern, expression of Cx43 at day 3 culture was higher for A1-, A2 and S1/PLGA scaffolds. This is consistent with literature showing important role of Osx in Cx43 expression [44, 45]. Importantly, we also observed relatively higher levels of Cx43 for cells cultured on the scaffolds without P_2_O_5_. Culturing cells for 7 days resulted in some increases in Cx43 expression for high-silica materials containing P_2_O_5_ (i.e. S2/PLGA), suggesting that in longer cultures phosphorus content in the high-silica scaffold may promote cell-cell communications. Overall, examination of typical osteoblast markers showed significant increases of collagen type I and osteonectin mRNA at day 7 culture, osteocalcin and bone sialoprotein at culture day 21 for A1/PLGA. Thus, these high-calcium composites without P_2_O_5_ seemed the best ones to induce and maintain osteogenesis. Although osteonectin and osteopontin mRNAs were elevated on silica-enriched composites, expression of all other markers on these composites was close to that on PLGA. Though, P_2_O_5_ presence in S2/PLGA composites were probably beneficial to induce the highest ALP activity in hBMSC.

Ectopic bone formation is a standard procedure to verify osteoinductivity of materials [30]. Previously, we examined osteoinductive potential of selected scaffolds of SiO_2_–CaO–P_2_O_5_ system, namely A2/PLGA and S2/PLGA composites, and both scaffolds were more effective at inducing ectopic bone formation in a rabbit muscle compared to plain PLGA [27]. Giving that our in present in vitro studies the scaffolds depleted of P_2_O_5_, i.e. A1/PLGA were the most effective at inducting hBMSC osteogenesis, it was plausible to verify its osteogenic potential in vivo along with the other studied S1/PLGA composite containing bioactive glass of SiO_2_–CaO system. When implanted, both A1/PLGA and S1/PLGA composite scaffolds performed well without causing any adverse tissue effects, but the morphometric data indicated more osteoid formation upon A1/PLGA implantation, which correlated well with the data obtained in vitro for this scaffold type. Altogether, in this work the PLGA-based scaffolds enriched with binary SiO_2_–CaO bioactive glasses emerge as promising candidates for bone tissue engineering and bone regeneration-related applications, especially that they may attract osteoprogenitor cells and drive their osteogenesis without additional pharmacological treatment. Moreover, P_2_O_5_ addition to bioactive glass composition significantly affects in vitro hBMSC responses, based on this and our previous study [24]. Thus one may expect different outcomes upon their long-term implantation. Direct comparison of the in vivo effects of bioactive glasses enriched or depleted of P_2_O_5_ are necessary and this will be a subject of our further studies.

## Conclusions

In this work we show that is possible to achieve a complete osteogenesis on SBG/PLGA porous scaffolds without any additional pharmacological treatment, but the in vitro and in vivo effects are strongly dependent on bioactive glass CaO/SiO_2_ ratio and the presence/absence of P_2_O_5_. The biological criteria of scaffolds evaluation and selection for in vivo implantation were based not only on the expression of typical osteogenic markers in osteoprogenitor cells, but on the scaffolds potential to increase osteoprogenitor cells migration rate and activate in them the endogenous expression and intracellular BMP signaling. Based on such evaluation scheme, we determined that the presence of P_2_O_5_ in the scaffolds negatively influenced not only the expression of osteogenic transcription factors Runx2 and Osx, but also cell-cell interactions as revealed by lower Cx43 expression in hBMSC cultured on P_2_O_5_-enriched composites. Moreover, the scaffolds of the highest CaO/SiO_2_ ratio without P_2_O_5_ were the only ones capable to increase hBMSC migration, activate BMP promoter in BRITE cells and induce both canonical and non-canonical BMP-related intracellular signaling in hBMSC. These scaffolds also produced higher amounts of osteoid in ectopic bone formation studies vs. their respective low calcium/high silica S1/PLGA counterpart. Thus, the potential of bioactive material scaffolds to provide, at early culture points, good cell-cell communication, increase osteoprogenitor cells migration and activate BMP-related multiple signaling pathways may constitute a new set of molecular markers to predict osteoinductive properties of studied implant materials.

## Data Availability

Not applicable.
